# Well-posedness for a class of generalized variational-hemivariational inequalities involving set-valued operators

**DOI:** 10.1186/s13660-018-1776-x

**Published:** 2018-07-24

**Authors:** Caijing Jiang

**Affiliations:** College of Sciences, Guangxi University for Nationalities, Nanning, P.R. China

**Keywords:** Generalized variational-hemivariational inequality, Set-valued operator, *α*-well-posedness, Monotonicity

## Abstract

The aim of present work is to study some kinds of well-posedness for a class of generalized variational-hemivariational inequality problems involving set-valued operators. Some systematic approaches are presented to establish some equivalence theorems between several classes of well-posedness for the inequality problems and some corresponding metric characterizations, which generalize many known results. Finally, the well-posedness for a class of generalized mixed equilibrium problems is also considered.

## Introduction

Nowadays, well-posedness has been drawing great attention in the field of optimization problems and related problems such as variational inequalities, hemivariational inequalities, fixed point problems, equilibrium problems, and inclusion problems (see [1, 5, 9, 11, 17, 19, 21, 23, 33]). The classical concept of well-posedness for a global minimization problem was first introduced by Tikhonov [35], which required the existence and uniqueness of a solution to the global minimization problem and the convergence of every minimizing sequence toward the unique solution. Thereafter, the concept of well-posedness has been generalized to variational inequalities. The initial notion of well-posedness for variational inequality is due to Lucchetti and Patrone [28]. Fang [13, 14] generalized two kinds of well-posedness for a mixed variational inequality problem in a Banach space. For further results on the well-posedness of variational inequalities, we refer to [2, 4, 12–14, 16, 22, 27, 28] and the references therein.

As an important and useful generalization of variational inequality, hemivariational inequality, which was first studied by Panagiotopoulos [32], has a great development in recent years by several works [6, 29, 31]. Many authors are interested in generalizing the concept of well-posedness to hemivariational inequalities. In 1995, Goeleven and Mentagui [15] generalized the concept of the well-posedness to a hemivariational inequality and presented some basic results concerning the well-posed hemivariational inequality. Recently, using the concept of approximating sequence, Xiao et al. [37, 38] introduced a concept of well-posedness for a hemivariational inequality and a variational-hemivariational inequality. Ceng, Lur, and Wen [3] considered an extension of well-posedness for a minimization problem to a class of generalized variational-hemivariational inequalities with perturbations in reflexive Banach spaces. For more recent works on the well-posedness for variational-hemivariational inequalities, we refer to [3, 15, 18, 19, 26, 37, 38] and the references therein.

In the last years, many authors studied the existence results for some types of hemivariational inequalities involving set-valued operators [34, 36, 39]. In 2011, Zhang and He [39] studied a kind of hemivariational inequalities of Hartman–Stampacchia type by introducing the concept of stable quasimonotonicity. They supposed that the constraint set is a bounded (or unbounded), closed, and convex subset in a reflexive Banach space. The authors gave sufficient conditions for the existence and boundedness of solutions. In 2013, Tang and Huang [34] generalized the result of [39] by introducing the concept of stable *ϕ*-quasimonotonicity and obtained some existence theorems when the constrained set is nonempty, bounded (or unbounded), closed, and convex in a reflexive Banach space. Hereafter, Wangkeeree and Preechasilp [36] generalized the results of [34] and [39] by introducing the concept of stable *f*-quasimonotonicity. Very recently, Liu and Zeng obtained some existence results for a class of hemivariational inequalities involving the stable $(g,f,\alpha)$-quasimonotonicity [25], a result on the well-posedness for mixed quasivariational hemivariational inequalities [26], and some existence results for a class of quasimixed equilibrium problems involving the $(f,g,h)$-quasimonotonicity [24].

Let *K* be a nonempty, closed, and convex subset of a real Banach space *X* with its dual $X^{*}$, and let $F:K\rightarrow P(X^{*})$ be a set-valued operator, where $P(X^{*})$ is the set of all nonempty subsets of $X^{*}$. Let $T:K\rightarrow X^{*}$ be a perturbation, and let $f\in X^{*}$ be a given element. Let $g:K\times K\rightarrow \overline{R}:=R\cup \{\pm \infty \}$ be a function such that $\mathcal{D}(g)=\{u\in K:g(u,v)\neq -\infty, \forall v\in K\}\neq \emptyset $. Let $J:X\to R$ be a locally Lipschitz function, and let $J^{\circ }(u,v)$ denote the generalized directional derivative in the sense of Clarke of a locally Lipschitz functional $J:X\rightarrow R$ at *u* in the direction *v*. In this paper, we discuss the following generalized variational-hemivariational inequality (GVHVI):

Find $u\in K$ such that, for some $u^{*}\in F(u)$,

$$\begin{aligned} \bigl\langle u^{*}+ Tu-f,v-u\bigr\rangle +g(u,v)+J^{\circ }(u;v-u) \geq 0, \quad \forall v\in K. \end{aligned}$$

Now, let us consider some particular cases of GVHVI.

- If $T\equiv 0$, $f\equiv 0$, and $g\equiv 0$, then GVHVI is reduced to the following form:
  Find $u\in K$ and $u^{*}\in F(u)$ such that
  $$\begin{aligned} \bigl\langle u^{*},v-u\bigr\rangle +J^{\circ }(u;v-u)\geq 0, \quad \forall v\in K. \end{aligned}$$
  The existence of solutions to this inequality was recently studied by Zhang and He [39].
- If $T\equiv 0$ and $f\equiv 0$, and $g(u,v)=\phi (v)-\phi (u)$ for all $u,v\in K$, then GVHVI is reduced to the following form:
  Find $u\in K$ and $u^{*}\in F(u)$ such that
  $$\begin{aligned} \bigl\langle u^{*},v-u\bigr\rangle +\phi (v)-\phi (u)+J^{\circ }(u;v-u) \geq 0, \quad \forall v\in K. \end{aligned}$$
  The existence of solutions to this inequality was studied by Tang and Huang [34].
- If $T\equiv 0$ and $f\equiv 0$, then GVHVI is reduced to the following form:
  Find $u\in K$ and $u^{*}\in F(u)$ such that
  $$\begin{aligned} \bigl\langle u^{*},v-u\bigr\rangle +g(u,v)+J^{\circ }(u;v-u)\geq 0, \quad \forall v\in K. \end{aligned}$$

The existence of solutions to this inequality was studied by Wangkeeree and Preechasilp [36].

Inspired by previous works, we study the well-posedness for GVHVI, which generalizes many known works. Under relatively weak conditions, we establish some equivalence results and some metric characterizations for the strong and weak *α*-well-posed GVHVI in the generalized sense. In particular, we present equivalence results on weak *α*-well-posedness for GVHVI, which were considered by few authors.

This paper is organized as follows. In Sect. 2, we recall some basic preliminaries of single-valued and set-valued mappings, metric concepts, Clarke’s generalized directional derivative, and some classes of well-posedness for GVHVI. In Sect. 3, we show some equivalence results for the well-posedness for GVHVI and some corresponding metric characterizations. Theorems 3.3, 3.5, and 3.6 are the main results in this section. In the last section, we also present the well-posedness for a class of generalized mixed equilibrium problems.

## Preliminaries

Let *R*, $R_{+}$, and *N* be the sets of real numbers, nonnegative real numbers, and natural numbers, respectively. Let *X* be a real Banach space with norm $\|\cdot \|_{X}$. Denote by $X^{*}$ its dual space and by $\langle \cdot,\cdot \rangle_{X}$ the duality pairing between $X^{*}$ and *X*. Let $X_{w}$ be the Banach space *X* with weak topology.

### Definition 2.1

Let *K* be a nonempty subset of *X*. A function $f:K\rightarrow R$ is said to be

(i) convex on *K* if for all finite subsets $\{u_{1},\ldots,u_{n} \}\subset K$ and $\{\lambda_{1},\ldots,\lambda_{n}\}\subset R_{+}$ such that $\sum_{i=1}^{n}\lambda_{i}=1$ and $\sum_{i=1}^{n}\lambda_{i}u _{i}\in K$, we have
  $$\begin{aligned} f\Biggl(\sum_{i=1}^{n}\lambda_{i}u_{i} \Biggr)\leq \sum_{i=1}^{n} \lambda_{i}f(u _{i}); \end{aligned}$$
(ii) (weakly) upper semicontinuous (u.s.c. for short) at *u* if for any sequence $\{u_{n}\}_{n\geq 1}\subset K$ with ($u_{n}\rightharpoonup u$) $u_{n}\rightarrow u$, we have
  $$\begin{aligned} \limsup_{n\rightarrow \infty }f(u_{n})\leq f(u). \end{aligned}$$
(iii) (weakly) lower semicontinuous (l.s.c. for short) at *u*, if for any sequence $\{u_{n}\}_{n\geq 1}\subset K$ with ($u_{n}\rightharpoonup u$) $u_{n}\rightarrow u$, we have
  $$\begin{aligned} \liminf_{n\rightarrow \infty }f(u_{n})\geq f(u). \end{aligned}$$
  The function *f* is said to be (weakly) u.s.c. (l.s.c.) on *K* if *f* is (weakly) u.s.c. (l.s.c.) at all $u\in K$.

### Definition 2.2

([20])

Let *K* be a nonempty subset of *X*. An operator $\beta:K\rightarrow X$ is said to be affine if for any $u_{i}\in K$ ($i=1,2,\ldots,n$) and $\lambda_{i}\in [0,1]$ with $\sum_{i=1} ^{n}\lambda_{i}=1$, we have

$$\begin{aligned} \beta \Biggl(\sum_{i=1}^{n} \lambda_{i}v_{i}\Biggr)=\sum_{i=1}^{n} \lambda_{i}\beta (u_{i}). \end{aligned}$$

### Definition 2.3

A set-valued operator $F:K\rightarrow P(X^{*})$ is said to be

(i) lower semicontinuous (l.s.c.) at $u_{0}$ if for any $u_{0}^{*}\in F(u_{0})$ and sequence $\{u_{n}\}_{n\geq 1}\subset K$ with $u_{n}\rightarrow u_{0}$, there exists a sequence $u_{n}^{*}\in F(u _{n})$ that converges to $u_{0}^{*}$.
(ii) lower hemicontinuous (l.h.c.) if the restriction of *F* to every line segment of *K* is lower semicontinuous with respect to the weak topology in $X^{*}$.

### Definition 2.4

A set-valued operator $F:K\rightarrow P(X^{*})$ is said to be monotone if for all $u,v\in K$,

$$\begin{aligned} \bigl\langle v^{*}-u^{*},v-u\bigr\rangle \geq 0, \quad \forall u^{*}\in F(u),\forall v^{*}\in F(v). \end{aligned}$$

### Definition 2.5

Let *S* be a nonempty subset of *X*. The measure *μ* of noncompactness for the set *S* is defined by

$$\begin{aligned} \mu (S):=\inf \Biggl\{ \epsilon >0:S=\bigcup_{i=1} ^{n} S_{i}, \operatorname{diam} \vert S_{i} \vert < \epsilon,i=1,2,\ldots,n\Biggr\} , \end{aligned}$$

where diam$|S_{i}|$ is the diameter of the set $S_{i}$.

Now, let us recall the definitions of the Clarke generalized directional derivative and generalized gradient for a locally Lipschitz function $\varphi:X\rightarrow R$ (see [6, 10]). The Clarke generalized directional derivative $\varphi^{0}(u;v)$ of *φ* at the point $u\in X$ in the direction $v\in X$ is defined as

$$ \varphi^{0}(u;v):=\limsup_{\lambda \rightarrow 0^{+},\zeta \rightarrow u}\frac{\varphi (\zeta +\lambda v)-\varphi (\zeta)}{\lambda }. $$

The Clarke subdifferential or generalized gradient of *φ* at $u\in X$, denoted by $\partial \varphi (u)$, is the subset of $X^{*}$ given by

$$ \partial \varphi (u):=\bigl\{ u^{*}\in X^{*}: \varphi^{0}(u;v)\geq \bigl\langle u^{*},v\bigr\rangle _{X}, \forall v\in X\bigr\} . $$

### Lemma 2.6

([6], Proposition 2.1.1)

*Let* $\varphi:X\rightarrow R$ *be locally Lipschitz of rank* $L_{u}>0$ *near u*. *Then*

(i) $\varphi^{0}(u;v)$ *is u*.*s*.*c*. *as a function of* $(u,v)$ *and*, *as a function of* *v* *alone*, *is Lipschitz of rank* $L_{u}$ *near* *u* *on* *X* *and satisfies*
  $$ \bigl\vert \varphi^{0}(u;v) \bigr\vert \leq L_{u} \Vert v \Vert _{X}; $$
(ii) *the gradient* $\partial \varphi (u)$ *is a nonempty*, *convex*, *and weakly*^∗^ *compact subset of* $X^{*}$ *bounded by a Lipschitz constant* $L_{u}$ *near* *x*;
(iii) *for every* $v\in X$, *we have*
  $$ \varphi^{0}(u;v)= \max \bigl\{ \bigl\langle u^{*},v\bigr\rangle |u^{*}\in \partial \varphi (u)\bigr\} . $$

We end this section with the notions of several classes of *α*-approximating sequences and *α*-well-posedness for GVHVI. Let $\alpha:X\to R_{+}$ be a functional.

### Definition 2.7

A sequence $\{u_{n}\}$ in *K* is an *α*-approximating sequence for GVHVI if there exist $\{u^{*}_{n}\}$ in $X^{*}$ with $u^{*}_{n}\in F(u _{n})$ and a nonnegative sequence $\{\epsilon_{n}\}$ with $\epsilon _{n}\rightarrow 0$ as $n\rightarrow \infty $ such that, for every $n\in N$,

$$\begin{aligned} \bigl\langle u_{n}^{*}+ Tu_{n}-f,v-u_{n} \bigr\rangle +g(u_{n},v)+J^{\circ }(u _{n};v-u_{n}) \geq -\epsilon_{n}\alpha (v-u_{n}),\quad \forall v\in K. \end{aligned}$$

In particular, if $\alpha (\cdot) = \|\cdot \|_{X}$, then $\{u_{n}\}$ is said to be an approximating sequence for GVHVI.

### Definition 2.8

GVHVI is said to be strongly (respectively, weakly) *α*-well-posed if it has a unique solution *u* and every *α*-approximating sequence $\{u_{n}\}$ strongly (respectively, weakly) converges to *u*. In particular, if $\alpha (\cdot) = \|\cdot \|_{X}$, then GVHVI is said to be strongly (respectively, weakly) well-posed.

### Definition 2.9

GVHVI is said to be strongly (respectively, weakly) *α*-well-posed in the generalized sense if the solution set Γ of GVHVI is nonempty and every *α*-approximating sequence $\{u_{n}\}$ has a subsequence that strongly (respectively, weakly) converges to some point of Γ. In particular, if $\alpha (\cdot) = \|\cdot \|_{X}$, then GVHVI is said to be strongly (respectively, weakly) well-posed in the generalized sense.

### Remark 2.10

Strong *α*-well-posedness (in the generalized sense) implies weak *α*-well-posedness (in the generalized sense), but the converse is not true in general.

## The characterizations of well-posedness for GVHVI

In this section, we establish metric characterizations and derive some conditions under which GVHVI is strongly (weakly) *α*-well-posed.

For any $\epsilon >0$, we define the following two sets:

$$\begin{aligned} \Omega_{\alpha }(\epsilon) =&\bigl\{ u\in K: \exists u^{*}\in F(u)\mbox{ such that }\bigl\langle u^{*}+ Tu-f,v-u\bigr\rangle +g(u,v) \\ & {} +J^{\circ }(u;v-u)\geq -\epsilon \alpha (v-u), \forall v \in K \bigr\} \end{aligned}$$

and

$$\begin{aligned} \Phi_{\alpha }(\epsilon) =&\bigl\{ u\in K:\bigl\langle v^{*}+Tu-f,v-u \bigr\rangle +g(u,v)+J ^{\circ }(u;v-u) \\ & {}\geq -\epsilon \alpha (v-u), \forall v\in K,\forall v^{*} \in F(v) \bigr\} . \end{aligned}$$

Denote by Γ the set of solutions to GVHVI. It is clear that $\Gamma =\Omega_{0}(\epsilon)$.

### Lemma 3.1

*Assume that*:

(i) *K* *is a nonempty closed subset of a real Banach space* *X*;
(ii) $T:K \rightarrow X^{*}_{w}$ *is continuous*;
(iii) $g:K\times K\rightarrow R$ *is u*.*s*.*c*. *with respect to the first variable*;
(iv) $\alpha:X\to R_{+}$ *is such that* $\liminf_{n\rightarrow \infty }\alpha (v_{n})\le \alpha (v)$ *whenever* $v_{n}\rightarrow v$.

*Then*, *for every* $\epsilon >0$, *the set* $\Phi_{\alpha }(\epsilon)$ *is closed in* *X*.

### Proof

Let $\{u_{n}\}\subset \Phi_{\alpha }(\epsilon)$ be s sequence such that $u_{n} \rightarrow u$ in *X*. Then $u\in K$, and, for all $v\in K$ and $v^{*}\in F(v)$,

$$\begin{aligned} \bigl\langle v^{*}+ Tu_{n}-f,v-u_{n}\bigr\rangle +g(u_{n},v)+J^{\circ }(u_{n};v-u _{n})\geq - \epsilon \alpha (v-u_{n}). \end{aligned}$$

By the assumptions and the properties of $J^{\circ }$ we have

$$\begin{aligned}& \bigl\langle v^{*}+ Tu-f,v-u\bigr\rangle +g(u,v)+J^{\circ }(u;v-u) \\& \quad \geq \limsup_{n\rightarrow \infty }\bigl[\bigl\langle v^{*}+ Tu_{n}-f,v-u_{n} \bigr\rangle +g(u_{n},v)+J^{\circ }(u_{n};v-u_{n}) \bigr] \\& \quad \geq \limsup_{n\rightarrow \infty }-\epsilon \alpha (v-u_{n}) \\& \quad =-\epsilon \liminf_{n\rightarrow \infty }\alpha (v-u_{n}) \\& \quad \ge -\epsilon \alpha (v-u), \end{aligned}$$

and hence

$$\begin{aligned} \bigl\langle v^{*}+ Tu-f,v-u\bigr\rangle +g(u,v)+J^{\circ }(u;v-u)- \epsilon \alpha (v-u), \quad \forall v\in K,\forall v^{*}\in F(v), \end{aligned}$$

which shows that $u\in \Phi_{\alpha }(\epsilon)$. □

### Lemma 3.2

*Assume that*:

(i) *K* *is a nonempty convex subset of a real Banach space* *X*;
(ii) $F:K\rightarrow P(X^{*})$ *is l*.*h*.*c*. *and monotone*;
(iii) $g:K\times K\rightarrow R$ *is convex with respect to the second variable*;
(iv) $\alpha:X\to R_{+}$ *is convex with* $\alpha (tv)=t\alpha (v)$ *for all* $t\ge 0$ *and* $v\in X$.

*Then* $\Omega_{\alpha }(\epsilon)=\Phi_{\alpha }(\epsilon)$ *for all* $\epsilon >0$.

### Proof

We first show that $\Omega_{\alpha }(\epsilon)\subset \Phi_{\alpha }( \epsilon)$. Indeed, take arbitrary $u\in \Omega_{\alpha }(\epsilon)$. Then there exists $u^{*}\in F(u)$ such that

$$\begin{aligned} \bigl\langle u^{*}+ Tu-f,v-u\bigr\rangle +g(u,v)+J^{\circ }(u;v-u) \geq -\epsilon \alpha (v-u), \quad \forall v\in K. \end{aligned}$$

According to the monotonicity of *F*, we obtain

$$\begin{aligned} \bigl\langle v^{*}+ Tu-f,v-u\bigr\rangle +g(u,v)+J^{\circ }(u;v-u) \geq -\epsilon \alpha (v-u), \quad \forall v\in K,\forall v^{*}\in F(v), \end{aligned}$$

which means that $u\in \Phi_{\alpha }(\epsilon)$. Therefore $\Omega_{\alpha }(\epsilon)\subset \Phi_{\alpha }(\epsilon)$.

Now we show that $\Phi_{\alpha }(\epsilon)\subset \Omega_{\alpha }( \epsilon)$. Indeed, take arbitrary $u\in \Phi_{\alpha }(\epsilon)$. Then

$$\begin{aligned} \bigl\langle v^{*}+ Tu-f,v-u\bigr\rangle +g(u,v)+J^{\circ }(u;v-u) \geq -\epsilon \alpha (v-u), \quad \forall v\in K,\forall v^{*}\in F(v). \end{aligned}$$

Since the set *K* is convex, for any $v\in K$ and $\lambda \in [0,1]$, taking $v_{\lambda }:=\lambda v+(1-\lambda)u\in K$ in this inequality, we have

$$\begin{aligned} \bigl\langle v_{\lambda }^{*}+ Tu-f,v_{\lambda }-u\bigr\rangle +g(u,v_{\lambda })+J^{\circ }(u;v_{\lambda }-u) \geq -\epsilon \alpha (v_{\lambda }-u), \quad \forall v_{\lambda }^{*}\in F(v_{\lambda }). \end{aligned}$$

Then by (iii), (iv), and the properties of $J^{\circ }$ we obtain

3.1 $$\begin{aligned} \bigl\langle v_{\lambda }^{*}+ Tu-f,v-u\bigr\rangle +g(u,v)+J^{\circ }(u;v-u) \geq -\epsilon \alpha (v-u), \quad \forall v_{\lambda }^{*}\in F(v_{\lambda }). \end{aligned}$$

Let $u^{*}\in F(u)$ be fixed, and let $v_{\lambda }^{*}\in F(v_{ \lambda })$ be such that $v_{\lambda }^{*}\rightharpoonup u^{*}$ in $X^{*}$ (the existence of such a sequence is ensured by the fact that *F* is l.h.c.). Taking the limit as $\lambda \rightarrow 0$ in (3.1), we obtain

$$\begin{aligned}& \bigl\langle u^{*}+ Tu-f,v-u\bigr\rangle +g(u,v)+J^{\circ }(u;v-u) \\& \quad =\lim_{\lambda \rightarrow 0}\bigl[\bigl\langle v_{\lambda }^{*}+ Tu-f,v-u \bigr\rangle +g(u,v)+J^{\circ }(u;v-u)\bigr] \\& \quad \geq -\epsilon \alpha (v-u), \end{aligned}$$

which implies that $u\in \Omega_{\alpha }(\epsilon)$. The proof is complete. □

The following result is a consequence of Lemmas 3.1 and 3.2.

### Theorem 3.3

*Assume that*:

(i) *K* *is a nonempty closed convex subset of a real Banach space* *X*;
(ii) $F:K\rightarrow P(X^{*})$ *is l*.*h*.*c*. *and monotone*;
(iii) $T:K\rightarrow X^{*}_{w}$ *is continuous*;
(iv) $g:K\times K\rightarrow R$ *is u*.*s*.*c*. *with respect to the first variable and convex with respect to the second variable*;
(v) $\alpha:X\to R_{+}$ *is continuous and convex with* $\alpha (tv)=t \alpha (v)$ *for all* $t\ge 0$ *and* $v\in X$.

*Then* $\Omega_{\alpha }(\epsilon)=\Phi_{\alpha }(\epsilon)$ *is closed in* *X* *for all* $\epsilon >0$. *Moreover*, $\Gamma =\Omega_{0}(\epsilon)=\Phi_{0}(\epsilon)$, *that is*, *GVHVI is equivalent to the following problem*:

*Find* $u\in K$ *such that*

$$\begin{aligned} \bigl\langle v^{*}+ Tu-f,v-u\bigr\rangle +g(u,v)+J^{\circ }(u;v-u) \geq 0, \quad \forall v\in K,v^{*}\in F(v). \end{aligned}$$

### Theorem 3.4

*GVHVI is strongly* *α*-*well*-*posed if and only if* Γ *is nonempty and*

$$\begin{aligned} \lim_{\epsilon \rightarrow 0}\operatorname{diam}\bigl( \Omega_{\alpha }( \epsilon)\bigr)=0. \end{aligned}$$

### Proof

The proof is similar to that of Theorem 4.3 in [26] by the assumptions of *g*. □

### Theorem 3.5

*Assume that all the assumptions of Theorem* 3.3 *are satisfied*. *Then GVHVI is strongly* *α*-*well*-*posed if and only if*

3.2 $$\begin{aligned} \Omega_{\alpha }(\epsilon)\neq \emptyset \quad \forall \epsilon \geq 0 \quad \textit{and}\quad \lim_{\epsilon \rightarrow 0} \operatorname{diam}\bigl( \Omega_{\alpha }(\epsilon)\bigr)=0. \end{aligned}$$

### Proof

Suppose that GVHVI is strongly *α*-well-posed. Then GVHVI has a unique solution $u\in K$, and thus $\Gamma \neq \emptyset $. Now, we prove that (3.2) holds. Clearly, $\Omega_{\alpha }(\epsilon) \supset \Gamma \neq \emptyset $. For the second part of (3.2), arguing by contradiction, let us assume that $\operatorname{diam}( \Omega_{\alpha }(\epsilon))$ does not tend to 0 as $\epsilon \rightarrow 0$. Thus for any nonnegative sequence $\{\epsilon_{n}\}$ with $\epsilon_{n}\rightarrow 0$ as $n\rightarrow \infty $, there exists a constant $\beta >0$ such that, for each $n\in N$, there exist $u_{n}^{(1)},u_{n}^{(2)}\in \Omega_{\alpha }(\epsilon_{n})$ satisfying

3.3 $$\begin{aligned} \bigl\Vert u_{n}^{(1)}-u_{n}^{(2)} \bigr\Vert >\beta >0. \end{aligned}$$

Since $u_{n}^{(1)},u_{n}^{(2)}\in \Omega_{\alpha }(\epsilon_{n})$, we know that the sequences $\{u_{n}^{(1)}\}$ and $\{u_{n}^{(2)}\}$ are both *α*-approximating sequences of GVHVI, and thus

3.4 $$\begin{aligned} \lim_{n\rightarrow }u_{n}^{(1)}=\lim _{n\rightarrow }u _{n}^{(2)}=u. \end{aligned}$$

From (3.3) and (3.4) we have

$$\begin{aligned} 0< \beta < \bigl\Vert u_{n}^{(1)}-u_{n}^{(2)} \bigr\Vert \leq \bigl\Vert u_{n}^{(1)}-u \bigr\Vert + \bigl\Vert u_{n}^{(2)}-u \bigr\Vert \rightarrow 0, \end{aligned}$$

which is a contradiction.

Conversely, assume that condition (3.2) holds. Let $\{u_{n}\}$ in *K* be an *α*-approximating sequence for GVHVI. Then, there exist $\{u^{*}_{n}\}$ in $X^{*}$ with $u_{n}^{*}\in F(u_{n})$ and a nonnegative sequence $\{\epsilon_{n}\}$ with $\epsilon_{n}\rightarrow 0$ as $n\rightarrow \infty $ such that, for every $n\in N$,

$$\begin{aligned} \bigl\langle u^{*}_{n}+ Tu_{n}-f,v-u_{n} \bigr\rangle +g(u_{n},v)+J^{\circ }(u _{n};v-u_{n}) \geq -\epsilon_{n}\alpha (v-u_{n}), \quad \forall v\in K, \end{aligned}$$

that is, $u_{n}\in \Omega_{\alpha }(\epsilon_{n})$ for all $n\in N$. By condition (3.2) we deduce that the sequence $\{u_{n}\}$ is a Cauchy sequence, and so $\{u_{n}\}$ converges strongly to some point $u\in K$. Let us show that $u\in K$ is a solution for GVHVI. By the monotonicity of *F* we obtain that, for every $n\in N$,

$$\begin{aligned}& \bigl\langle v^{*}+ Tu_{n}-f,v-u_{n}\bigr\rangle +g(u_{n},v)+J^{\circ }(u _{n};v-u_{n}) \\& \quad \geq \bigl\langle u^{*}_{n}+Tu_{n}-f,v-u_{n} \bigr\rangle +g(u_{n},v)+J^{ \circ }(u_{n};v-u_{n}) \\& \quad \geq -\epsilon_{n}\alpha (v-u_{n}), \quad \forall v\in K,v^{*}\in F(v). \end{aligned}$$

By the assumptions we obtain that

$$\begin{aligned}& \bigl\langle v^{*}+Tu-f,v-u\bigr\rangle +g(u,v)+J^{\circ }(u;v-u) \\& \quad \geq \limsup_{n\rightarrow \infty }\bigl[\bigl\langle v^{*}+ Tu_{n}-f,v-u _{n}\bigr\rangle +g(u_{n},v)+J^{\circ }(u_{n};v-u_{n}) \bigr] \\& \quad \geq \limsup_{n\rightarrow \infty }-\epsilon_{n}\alpha (v-u _{n}) \\& \quad =\limsup_{n\rightarrow \infty }\alpha \bigl(-\epsilon_{n}(v-u_{n}) \bigr) \\& \quad =0, \end{aligned}$$

which implies that

$$\begin{aligned} \bigl\langle v^{*}+Tu-f,v-u\bigr\rangle +g(u,v)+J^{\circ }(u;v-u) \geq 0, \quad \forall v\in K,\forall v^{*}\in F(v). \end{aligned}$$

It follows from Theorem 3.3 that there exists $u^{*}\in F(u)$ such that

$$\begin{aligned} \bigl\langle u^{*}+ Tu-f,v-u\bigr\rangle +g(u,v)+J^{\circ }(u;v-u) \geq 0, \quad \forall v\in K. \end{aligned}$$

Then $u\in K$ is a solution of GVHVI.

Finally, we prove that the solution *u* is unique. If there exists another solution $u'\in K$, then $u,u_{1}\in \Omega_{\alpha }(\epsilon)$ for all $\epsilon >0$, and

$$\begin{aligned} 0< \bigl\Vert u-u' \bigr\Vert \leq \operatorname{diam}\bigl( \Omega_{\alpha }(\epsilon)\bigr)\rightarrow 0\quad \mbox{as }\epsilon \rightarrow 0, \end{aligned}$$

which is a contradiction. This completes the proof. □

### Theorem 3.6

*Assume that*:

(i) *K* *is a nonempty closed convex subset of a real reflexive Banach space* *X*;
(ii) $F:K\rightarrow P(X^{*})$ *is l*.*h*.*c*. *and monotone*;
(iii) $T:K\rightarrow X^{*}$ *is compact*;
(iv) $g:K\times K\rightarrow R$ *is weakly u*.*s*.*c*. *with respect to the first variable and convex with respect to the second variable*;
(v) $\limsup_{n\rightarrow \infty }J^{\circ }(u_{n};v-u_{n})\le J^{ \circ }(u;v-u)$ *for all* $v\in X$ *whenever* $u_{n}\rightharpoonup u$ *as* $n\rightarrow \infty $;
(vi) $\alpha:X\to R_{+}$ *is a continuous and convex functional with* $\alpha (tv)=t\alpha (v)$ *for all* $t\ge 0$ *and* $v\in X$.

*Then GVHVI is weakly* *α*-*well*-*posed if and only if GVHVI has a unique solution and there exists* $\epsilon_{0}>0$ *such that* $\Omega_{\alpha }(\epsilon_{0})$ *is nonempty and bounded*.

### Proof

The necessity is obvious. We now prove the sufficiency. Let $\{u_{n}\}$ be an *α*-approximating sequence for GVHVI. Then, there exist $\{u^{*}_{n}\}$ in $X^{*}$ with $u_{n}^{*}\in F(u_{n})$ and a nonnegative sequence $\{\epsilon_{n}\}$ with $\epsilon_{n}\rightarrow 0$ as $n\rightarrow \infty $ such that, for every $n\in N$,

$$\begin{aligned} \bigl\langle u^{*}_{n}+ Tu_{n}-f,v-u_{n} \bigr\rangle +g(u_{n},v)+J^{\circ }(u _{n};v-u_{n}) \geq -\epsilon_{n}\alpha (v-u_{n}) \end{aligned}$$

for all $v\in K$. We claim that the sequence $\{u_{n}\}$ is bounded in *X*. Indeed, since $\Omega_{\alpha }(\epsilon_{0})$ is bounded and $\Omega_{\alpha }(\epsilon)\subset \Omega_{\alpha }(\epsilon_{0})$ for all $\epsilon \in (0,\varepsilon_{0})$, there exists $n_{0}\in N$ such that $\epsilon_{n_{0}}\in (0,\varepsilon_{0})$ and $u_{n}\in \Omega_{\alpha }(\epsilon_{0})$ for all $n\ge n_{0}$, which shows that $\{u_{n}\}$ is bounded in *X*.

Since the Banach space *X* is reflexive, we can choose a subsequence of $\{u_{n}\}$, denoted by $\{u_{n}\}$ again, such that $u_{n}\rightharpoonup \overline{u}$ as $n\rightarrow \infty $ for some $\overline{u}\in X$. Let us show that $\overline{u}\in K$ is a solution for GVHVI. Obviously, $\overline{u}\in K$. By the monotonicity of *F* we obtain that

$$\begin{aligned}& \bigl\langle v^{*}+ Tu_{n}-f,v-u_{n}\bigr\rangle +g(u_{n},v)+J^{\circ }(u _{n};v-u_{n}) \\& \quad \geq \bigl\langle u^{*}_{n}+Tu_{n}-f,v-u_{n} \bigr\rangle +g(u_{n},v)+J^{ \circ }(u_{n};v-u_{n}) \\& \quad \geq -\epsilon_{n}\alpha (v-u_{n}),\quad \forall v\in K,v^{*}\in F(v), \forall n\in N. \end{aligned}$$

By the assumptions, we obtain that

$$\begin{aligned}& \bigl\langle v^{*}+T\overline{u}-f,v-\overline{u}\bigr\rangle +g( \overline{u},v)+J^{\circ }(\overline{u};v-\overline{u}) \\& \quad \geq \limsup_{n\rightarrow \infty }\bigl[\bigl\langle v^{*}+ Tu_{n}-f,v-u _{n}\bigr\rangle +g(u_{n},v)+J^{\circ }(u_{n};v-u_{n}) \bigr] \\& \quad \geq \limsup_{n\rightarrow \infty }-\epsilon_{n}\alpha (v-u _{n}) \\& \quad =\limsup_{n\rightarrow \infty }\alpha \bigl(-\epsilon_{n}(v-u_{n}) \bigr) \\& \quad =0, \end{aligned}$$

which implies that

$$\begin{aligned} \bigl\langle v^{*}+T\overline{u}-f,v-\overline{u}\bigr\rangle +g( \overline{u},v)+J ^{\circ }(\overline{u};v-\overline{u})\geq 0, \quad \forall v\in K,\forall v^{*}\in F(v). \end{aligned}$$

It follows from Theorem 3.3 that there exists $\overline{u}^{*}\in F(\overline{u})$ such that

$$\begin{aligned} \bigl\langle u^{*}+ T\,\overline{u}-f,v-\overline{u}\bigr\rangle +g( \overline{u},v)+J ^{\circ }(\overline{u};v-\overline{u})\geq 0, \quad \forall v\in K, \end{aligned}$$

Therefore $\overline{u}\in K$ is a solution to problem GVHVI, and so we get that GVHVI is weakly *α*-well-posed by the uniqueness of the solution to problem GVHVI. This completes the proof. □

### Remark 3.7

In the theorem, condition (v) can be found in [30], and the condition that there exists $\epsilon_{0}>0$ such that $\Omega_{ \alpha }(\epsilon_{0})$ is nonempty and bounded can be replaced by the conditions that *K* is bounded or that there exists $n_{0}\in N$ such that, for every $u\in K\setminus B_{n_{0}}$, there exists $v\in K$ with $\|v\|<\|u\|$ such that

$$\begin{aligned} \sup_{u^{*}\in F(u)}\bigl\langle u^{*}+Tu-f,v-u\bigr\rangle +g(u,v)+J^{\circ }(u;v-u) \leq -\frac{1}{n_{0}}. \end{aligned}$$

See [34, 36, 39] for more detail.

Next, we give some equivalence results for the strong *α*-posedness in the generalized sense.

### Theorem 3.8

*Assume that all the assumptions of Theorem* 3.5 *are satisfied*. *Then GVHVI is strongly* *α*-*well*-*posed in the generalized sense if and only if* Γ *is nonempty compact and*

$$\begin{aligned} \lim_{\epsilon \rightarrow 0}e\bigl(\Omega_{\alpha }( \epsilon),\Gamma\bigr)=0, \end{aligned}$$

*where* $e(A,B):=\sup_{a\in A}d(a,B)$ *with* $d(a,B):=\inf_{b\in B}\|a-b \|$.

### Proof

The proof is similar to that of Theorem 5.1 in [26] by the assumptions of *g*. □

### Theorem 3.9

*Assume that all the assumptions of Theorem* 3.5 *are satisfied*. *Then GVHVI is strongly* *α*-*well*-*posed in the generalized sense if and only if*

$$\begin{aligned} \Omega_{\alpha }(\epsilon)\neq \emptyset, \quad \forall \epsilon >0,\quad \textit{and}\quad \lim_{\epsilon \rightarrow 0}\mu \bigl(\Omega_{\alpha }( \epsilon)\bigr)=0. \end{aligned}$$

### Proof

The proof is similar to that of Theorem 3.2 in [3] by the assumptions of *g*. □

### Theorem 3.10

*Assume that all the assumptions of Theorem* 3.6 *are satisfied*. *Then GVHVI is weakly* *α*-*well*-*posed in the generalized sense if and only if there exists* $\epsilon_{0}>0$ *such that* $\Omega_{\alpha }(\epsilon_{0})$ *is nonempty and bounded*.

### Proof

The proof is similar to that of Theorem 3.6 by the assumptions of *g*. □

## Well-posedness for GMEP

In this section, we consider the following generalized mixed equilibrium problem (GMEP):

Find $u\in K$ such that, for some $u^{*}\in F(u)$,

$$\begin{aligned} \bigl\langle u^{*},\eta (u,v)\bigr\rangle +\langle Tu-f,v-u\rangle +g(u,v)+h(u,v) \geq 0, \quad \forall v\in K, \end{aligned}$$

where $\eta:K\times K\rightarrow X$ is an operator. The existence of solutions to this problem when $T\equiv 0$ and $f\equiv 0$ can be found in [25].

To study GMEP, we introduce the concept of *η*-monotonicity (see [7, 8]).

### Definition 4.1

Let $F:K\rightarrow P(X^{*})$ be a set-valued operator. *F* is said to be *η*-monotone if there exists a function $\eta:K\times K\rightarrow X$ such that, for all $u,v\in K$,

4.1 $$\begin{aligned} \bigl\langle v^{*}-u^{*},\eta (u,v)\bigr\rangle \geq 0, \quad \forall u^{*}\in F(u),\forall v^{*}\in F(v). \end{aligned}$$

### Remark 4.2

If $\eta (u,v)=v-u$ for all $u,v\in X$, then (4.1) becomes

$$\begin{aligned} \bigl\langle v^{*}-u^{*},v-u\bigr\rangle \geq 0, \quad \forall u^{*}\in F(u),\forall v^{*}\in F(v), \end{aligned}$$

that is, *F* is monotone.

For any $\epsilon >0$, we define the following two sets:

$$\begin{aligned} \Omega_{\eta,\alpha }(\epsilon) =&\bigl\{ u\in K: \exists u^{*}\in F(u)\mbox{ such that }\bigl\langle u^{*},\eta (u,v)\bigr\rangle + \langle Tu-f,v-u\rangle +g(u,v) \\ & {}+h(u,v))\geq -\epsilon \alpha (v-u),\forall v\in K \bigr\} \end{aligned}$$

and

$$\begin{aligned} \Phi_{\eta,\alpha }(\epsilon) =&\bigl\{ u\in K:\bigl\langle v^{*},\eta (u,v) \bigr\rangle +\langle Tu-f,v-u\rangle +g(u,v) \\ & {}+h(u,v)\geq -\epsilon \alpha (v-u), \forall v\in K,\forall v ^{*} \in F(v) \bigr\} . \end{aligned}$$

Denote by $\Gamma_{\eta }$ the set of solutions to GMEP. It is clear that $\Gamma =\Omega_{0}(\epsilon)$.

We can obtain similar results.

### Theorem 4.3

*Assume that all the assumptions of Theorem* 3.3 *are satisfied and*, *in addition*, $\eta:K\times K\rightarrow X$ *is continuous on* $K\times K$ *with* $\eta (u,u)=0$ *for any* $u\in K$ *and affine with respect to the first variable*. *Let* $h:K\times K\rightarrow R$ *be such that*:

(i) $h(u,u)=0$ *for all* $u\in X$,
(ii) *for all* $v\in K$, $h(\cdot,v)$ *is u*.*s*.*c*.,
(iii) *for all* $u\in K$, $h(u,\cdot)$ *is convex*.

*Then* $\Omega_{\eta,\alpha }(\epsilon)=\Phi_{\eta,\alpha }(\epsilon)$ *is closed in* *X* *for all* $\epsilon >0$. *Moreover*, $\Gamma_{\eta }= \Omega_{\eta,0}(\epsilon)=\Phi_{\eta,0}(\epsilon)$, *that is*, *GMEP is equivalent to the following problem*:

*Find* $u\in K$ *such that*

$$\begin{aligned} \bigl\langle v^{*}+ Tu-f,\eta (u,v)\bigr\rangle +g(u,v)+h(u,v)\geq 0, \quad \forall v\in K,v^{*}\in F(v). \end{aligned}$$

### Theorem 4.4

*Assume that all the assumptions of Theorem* 3.5 *are satisfied and*, *in addition*, $\eta:K\times K\rightarrow X$ *is continuous on* $K\times K$ *with* $\eta (u,u)=0$ *for any* $u\in K$ *and affine with respect to the first variable*. *Let* $h:K\times K\rightarrow R$ *be such that*:

(i) $h(u,u)=0$ *for all* $u\in X$,
(ii) *for all* $v\in K$, $h(\cdot,v)$ *is u*.*s*.*c*.,
(iii) *for all* $u\in K$, $h(u,\cdot)$ *is convex*.

*Then GMVHVI is strongly* *α*-*well*-*posed if and only if*

$$\begin{aligned} \Omega_{\eta,\alpha }(\epsilon)\neq \emptyset,\quad \forall \epsilon \geq 0, \quad \textit{and}\quad \lim_{\epsilon \rightarrow 0} \operatorname{diam}\bigl(\Omega_{\eta,\alpha }( \epsilon)\bigr)=0. \end{aligned}$$

### Theorem 4.5

*Assume that all the assumptions of Theorem* 3.6 *are satisfied and*, *in addition*, $\eta:K\times K\rightarrow X$ *is continuous on* $K\times K$ *with* $\eta (u,u)=0$ *for any* $u\in K$ *and affine with respect to the first variable*. *Let* $h:K\times K\rightarrow R$ *be such that*:

(i) $h(u,u)=0$ *for all* $u\in X$,
(ii) *for all* $v\in K$, $h(\cdot,v)$ *is weakly u*.*s*.*c*.,
(iii) *for all* $u\in K$, $h(u,\cdot)$ *is convex*.

*Then GMEP is weakly* *α*-*well*-*posed if and only if GMEP has a unique solution and there exists* $\epsilon_{0}>0$ *such that* $\Omega_{\alpha }(\epsilon_{0})$ *is nonempty and bounded*.

### Theorem 4.6

*Assume that all the assumptions of Theorem* 3.5 *are satisfied and*, *in addition*, $\eta:K\times K\rightarrow X$ *is continuous on* $K\times K$ *with* $\eta (u,u)=0$ *for any* $u\in K$ *and affine with respect to the first variable*. *Let* $h:K\times K\rightarrow R$ *is such that*:

(i) $h(u,u)=0$ *for all* $u\in X$,
(ii) *for all* $v\in K$, $h(\cdot,v)$ *is u*.*s*.*c*.,
(iii) *for all* $u\in K$, $h(u,\cdot)$ *is convex*.

*Then GMEP is strongly* *α*-*well*-*posed in the generalized sense if and only if*

$$\begin{aligned} \Omega_{\eta,\alpha }(\epsilon)\neq \emptyset, \quad \forall \epsilon >0,\quad \textit{and}\quad \lim_{\epsilon \rightarrow 0}\mu \bigl(\Omega_{\eta,\alpha }( \epsilon)\bigr)=0. \end{aligned}$$

### Theorem 4.7

*Assume that all the assumptions of Theorem* 3.6 *are satisfied and*, *in addition*, $\eta:K\times K\rightarrow X$ *is continuous on* $K\times K$ *with* $\eta (u,u)=0$ *for any* $u\in K$ *and affine with respect to the first variable*. *Let* $h:K\times K\rightarrow R$ *be such that*:

(i) $h(u,u)=0$ *for all* $u\in X$,
(ii) *for all* $v\in K$, $h(\cdot,v)$ *is weakly u*.*s*.*c*.,
(iii) *for all* $u\in K$, $h(u,\cdot)$ *is convex*.

*Then GMEP is weakly* *α*-*well*-*posed in the generalized sense if and only if there exists* $\epsilon_{0}>0$ *such that* $\Omega_{\alpha }( \epsilon_{0})$ *is nonempty and bounded*.

## Conclusion

In this paper, inspired by the previous works, we study the well-posedness for GVHVI. Under relatively weak conditions for the data *F*, *T*, *g*, *J* (see Theorems 3.3 and 3.6), we provide some equivalence results for the strong and weak *α*-well-posed GVHVI in the generalized sense. Our results generalize and improve many known results and can be applied to many other problems.
